# Political Orientation and Public Attributions for the Causes and Solutions of Physical Inactivity in Canada: Implications for Policy Support

**DOI:** 10.3389/fpubh.2019.00153

**Published:** 2019-06-18

**Authors:** Lira Yun, Leigh M. Vanderloo, Tanya R. Berry, Amy E. Latimer-Cheung, Norm O'Reilly, Ryan E. Rhodes, John C. Spence, Mark S. Tremblay, Guy Faulkner

**Affiliations:** ^1^School of Kinesiology, University of British Columbia, Vancouver, BC, Canada; ^2^ParticipACTION, Toronto, ON, Canada; ^3^Faculty of Kinesiology, Sport, and Recreation, University of Alberta, Edmonton, AB, Canada; ^4^School of Kinesiology and Health Studies, Queens University, Kingston, ON, Canada; ^5^College of Business and Economics, University of Guelph, Guelph, ON, Canada; ^6^School of Exercise Science, University of Victoria, Victoria, BC, Canada; ^7^Healthy Active Living and Obesity Research Group, Children's Hospital of Eastern Ontario Research Institute, Ottawa, ON, Canada

**Keywords:** physical activity, attribution, policy, political orientation, social climate

## Abstract

**Objectives:** To examine how public attributions for the causes and solutions of physical inactivity and individuals' self-identified political orientation are associated with support for different policy actions in addressing physical inactivity.

**Methods:** A secondary data analysis was conducted with a sample of 2,044 Canadian adults. Two sets of 2 X 3 analyses of variance and *post-hoc* analyses were conducted to assess (1) the mean differences by the causes of the issue of physical inactivity (individual, or both internal and external/external) and political orientation (liberal, centrist, and conservative), and (2) responsibility for solutions (private matter, or both private and public health matter, and /public health matter) and political orientation on support for least, moderate, and most intrusive policy actions.

**Results:** No interaction effects existed between causal attribution and political orientation on policy support, but a main effect of causal attributions for physical inactivity and political orientation was significant. Those who held internal attributions for the cause of physical inactivity showed less support for policies compared to those who held external causal attributions or both internal and external causal attributions. Conservative individuals reported the least support for all policy actions in comparison to liberal or centrist orientations. There were interaction effects between responsibility for solutions and political orientation on policy support. Conservative individuals who perceived the responsibility for solving physical inactivity as a private matter had less support for all three policy actions.

**Conclusions:** Public acceptance of policy actions addressing physical inactivity varies by the attributions the public have regarding causes and responsibility for solving the problem, and by political orientation. Advocacy and messaging for policy implementation in the physical activity arena needs to be communicated in ways that encourage reflective and informed deliberation that is representative of the Canadian population.

## Introduction

Physical activity has significant physical health benefits including prevention of non-communicable diseases (NCDs) such as cardiovascular disease, diabetes and cancer (1). It also provides cognitive and mental health benefits through a range of potential neurobiological, psychosocial, and behavioral processes (2). Nonetheless, the prevalence of physical inactivity in Canada and other developed countries is a serious public health concern that contributes, both directly and indirectly, to health care expenditures (3–5). For instance, the estimated global cost of physical inactivity in 2013 was 68 billion international dollars per year. Furthermore, inactivity accounts for 1–3% of national health care costs before costs associated with poor mental health and musculoskeletal conditions are considered (3, 4). Although extensive evidence supports the need for immediate institutional and government actions to re-create societies in which being physically active is the norm and socially desirable, little effort in implementing direct policy interventions (e.g., legislation and regulations to promote physical activity) has been taken to address physical inactivity at a population level (6). In contrast, sustained policy action has occurred on other public health matters and with considerable success. For example, tobacco control policies include implementing campaigns to increase awareness of the harms of smoking, taxing tobacco products, and executing legislations and regulations to ban smoking in public spaces, collectively leading to a reduction of smoking rates in the given population (7, 8). Obesity is currently one of the most serious public health issues in Canada and policies to encourage both healthy eating and physical activity have been implemented in that context (9, 10). Yet, little coordinated action has been conducted to address physical inactivity as an important modifiable risk factor in its own right irrespective of weight loss (11, 12).

In 2018, the World Health Organization released the “Global Action Plan on Physical Activity 2018-2030” to respond to the requests by countries for effective and feasible policies to increase physical activity (3). In Canada, the federal government released “A Common Vision for increasing physical activity and reducing sedentary living in Canada: Let's get moving” that describes a collective approach to policies, planning, and programming across Canada (13). In both documents, it is acknowledged that government interventions and policy actions across multiple departments and agencies including sport, recreation, health, culture, transportation, and education sectors will be necessary for creating active societies via changing social norms and attitudes. They also report creating active environments by changing spaces and places, creating active people via providing programs and opportunities, and creating active systems through governance and policy enablers, all of which contribute to success in addressing population-level physical inactivity. Before detailed planning, programs, and policies are developed and implemented, it is appropriate and timely to assess how much public support there is in Canada for different policy approaches. Understanding public opinion with regard to physical inactivity is essential because public resistance to policies may cause challenges in their implementation and even result in their withdrawal from consideration by policy makers (14).

One dimension by which policies may vary is in their degree of intrusiveness. This can be defined as “progressive steps from individual freedom and responsibility toward state intervention as one moves up the ladder (p. 42)” (15). The less intrusive approach is to simply monitor the situation or provide information while the most intrusive approach is to legislate in such a way that restricts individual freedom significantly in order to achieve gains in population health. For example, public education and communication campaigns to provide information on the importance of physical activity and harms of physical inactivity are relatively low in terms of their intrusiveness. Policies that are more intrusive include modifying community environments to provide more opportunities to participate in physical activity (e.g., building walking paths or bike lanes), guiding the choice through incentives for engaging in physical activity (e.g., providing tax credits for enrolling children in sport programs), and disincentives for not engaging in physical activity (e.g., higher life insurance premiums). The most intrusive approach is one that restricts or eliminates the choice of being physically inactive; for instance, legislation or regulations for *pedestrianization* of city centers or banning all traffic in high-use pedestrian areas during peak hours to support walking, cycling, or public transportation. Previous literature has demonstrated that public support generally decreases as the level of policy intrusiveness increases although intrusive policies generally have more impact on population behavior (16, 17). Particularly in western, liberal and democratic societies, such intrusive policy interventions may be unpopular as they run the risk of accusations of “nanny state-ism” where the government is seen as intruding “into the private lives of citizens and treats them as infants who cannot be trusted to make their own decisions (p. 1074)” (18). Therefore, the examination of how Canadians endorse different policy actions to address physical activity, from the least to most intrusive policy actions, would be helpful to inform future policy approaches and the implementation of the Common Vision (11).

Another factor influencing public support of different policy actions may be individuals' attribution of the cause and responsibility for solving a given health issue [c.f., attribution theory, (19, 20)]. That is, whether individuals attribute the causes of physical inactivity to something within one's control (internal) or outside of one's control (external) and whether the responsibility for solutions in addressing physical inactivity is a private matter that individuals need to deal with on their own or if it is a public health matter that requires governmental intervention. Previous research has revealed that how health issues are framed in terms of the attribution of the cause (internal vs. external) and responsibility for solutions (a private matter vs. a public health matter) impacts the acceptability of governmental intervention and support of policies (19). For example, people who hold internal causal attributions for obesity are more likely to perceive themselves responsible, whereas those who hold external causal attributions are more likely to support policies including more intrusive legislative and regulatory approaches (21–23). Though obesity and physical inactivity should not be confounded, one may surmise that the public is likely to support governmental interventions or policy actions if they perceive physical inactivity as a public health issue that is out of an individual's control. Thus, assessing attribution opinions for the causes of physical inactivity can provide guidance on how to approach and frame physical inactivity policy interventions.

Political orientation is another potential predictor of public support for public health policy initiatives. For instance, an individual's political orientation is associated with their views on responsibility for health and well-being (24); this, is further associated with the degree (extent) of support for different policy actions (25). In comparison to a conservative orientation, having a liberal orientation is more strongly associated with beliefs that society is responsible for one's fate and to support incremental social changes, whereas conservatives are more likely to believe that each individual is responsible for one's misfortune (26). This, in turn, negatively affects conservatives' attitudes toward government spending on health care and government-led policy actions (27). For instance, in a survey of Canadian federal and provincial/territorial legislators conducted in 2001, 36% of Liberal legislators agreed that government had a major role to play in encouraging people to be physically active. In contrast, 24% of the Progressive Conservative legislators endorsed this statement (28). Thus, examining the role of political orientation may help guide how to compose messages to promote public support of physical activity policies that resonate with the population regardless of political orientation, and help inform approaches for seeking government support tailored to current government orientation.

These issues have received little direct attention in the literature in the context of physical inactivity despite its prominence as a public health issue. Recent research reported an assessment of the social climate of physical inactivity in Canada exploring dimensions of the perceived seriousness of physical inactivity as a public health concern, social norms of physical activity, attributions for responsibility and solutions for solving inactivity, and acceptability of different policy approaches (29). The findings from that study indicated that physical inactivity was considered as a serious public health concern. Strong support existed for individual and economic level policies but much less for legislative approaches that are by definition more intrusive.

The present study extends the findings of the previous study (29) by examining how attributions for the causes and responsibility for solving physical inactivity are associated with support for different policy actions. Consistent with findings in the obesity context (30, 31), it was hypothesized that (1) perceptions of causes of physical inactivity would be associated with policy support such that those who attributed the cause of physical inactivity to the individual (internal cause) would be less likely to support all policy actions whereas those who held societal causal attributions (external cause) would be more supportive of the policy actions. In terms of responsibility for solving physical inactivity, it was hypothesized that (2) those who attributed the responsibility for solutions as a private matter would be less likely to support policy actions than those who attributed the responsibility as a public health matter. Furthermore, the study examines whether level of policy support, by attributions of the causes and responsibility for solutions, differs according to an individuals' political orientation. It was hypothesized that (3) individuals who self-identify as conservative and attribute physical inactivity to individual causes (and responsibility for solution as a private matter) would report less support for policy actions in comparison to individuals who identify as liberal or centrist. Different policy actions based on their level of intrusiveness were examined separately to explore whether the associations were (in)congruent across all levels of policy actions from low, moderate, to high intrusive policies.

## Methods

### Sample

ParticipACTION, a Canadian non-profit organization promoting physical activity (www.participaction.com), commissioned the original survey as part of its ongoing public relations and advocacy work. Specifically, a total sample of 2,519 participants were recruited from a representative sample of panelists (Canadian adults ≥18 years) drawn from an online panel collected via a Canadian survey firm (Angus Reid Forum) which includes 100,000 Canadians. The panel is considered comparable with the Canadian census in terms of age, sex, region, income, employment, and language spoken. The survey was deployed online in French and English on January 15, 2018 and remained open for seven days. By enrolling as a panelist in the Angus Reid Forum, recruited individuals consented to their participation in invited surveys or panel discussions. Given the secondary analyses undertaken in this study were of minimal risk and utilized anonymous data, ethical approval was not needed in accordance with article 2.4 and 5.5 of the Canadian Tri-Council policy statement (TCPS2) regarding ethical conduct of human research (32). All raw data were cleaned and de-identified before being delivered to the research team in a tabulated format using SPSS Version 23 (IBM, New York, USA).

### Measures

#### Support of Policies to Address Physical Inactivity

The primary dependent variables were support for policies to address physical inactivity. Physical inactivity is defined as not meeting national guidelines of recommended physical activity which is generally “at least 150 min of moderate-intensity physical activity per week”. In the study, physical activity was defined as “sports, fitness or recreational activities, organized or non-organized (e.g., gym exercise, cycling, running, and all team sports), active ways to get to places like walking or cycling, and any other physical activities while at work, in or around one's home or while volunteering”. Survey items were modified from previous studies assessing the social climate regarding obesity and tobacco control (33, 34) including the International Tobacco Control Survey (35) which assessed the following: individual responsibility for behaviors and modifying community environment; focusing on economic levers by providing incentives, subsidies, and tax credits; and targeting legislative changes to modify the environment and forces to discourage physical inactivity. After the following question: “People talk about many ways in which we could deal with physical inactivity in Canada. Which of these measures would you support and which would you oppose?” responses were made on a 7-point scale ranging from “strongly oppose (1)” to “strongly support (7)”. To determine if components needed to be treated separately or combined to best represent the data, a factor analysis (principal component analysis with Varimax rotation) was conducted. The results suggested a three-factor solution where each factor had an Eigenvalue >1. The three factors reflect groupings of policy actions based on the level of intrusiveness (low, moderately, most intrusive) with a total 54.6% of variance explained. Means of 10 items representing low intrusive policy (Cronbach's α = 0.87), of four items representing moderately intrusive policy (Cronbach's α = 0.74), and of three items representing the most intrusive policy (Cronbach's α = 0.65) were used as the dependent variables. All the policy items and the result of the factor analysis are presented as supplementary material (Supplementary Tables 1–3).

#### Attributions for the Causes of Physical Inactivity

Attributions for the cause of physical inactivity were measured in response to the probe “Which of the following statements best represent how you feel?” with one of the following options of physical inactivity being: “an individual's fault (internal cause),” “caused by other factors beyond an individual's control (external cause) ,” “both an individual's fault and caused by other factors beyond an individual's control (both internal and external cause),” “neither an individual's fault nor caused by other factors beyond an individual's control,” and “don't know.” Attributions for the causes of physical inactivity were re-coded to “internal cause” vs. “external cause and both internal and external cause.” Items were adapted from similar questions on obesity (33, 36).

#### Attributions for the Responsibility in Solving Physical Inactivity

Attribution for the responsibility in solving physical inactivity was assessed with a question adapted from the context of obesity (33, 36). After the probing question “Which of the following statements best represent how you feel?,” respondents were asked to choose one of the following options of physical inactivity being “a private matter that people need to deal with on their own (i.e., private matter),” “a public health matter that society needs to solve (i.e., public health matter),” “both a private matter and a public health matter,” “neither a private matter nor a public health matter,” and “don't know.” The responses were re-coded to “private matter” vs. “public health matter and both private and public health matter.”

#### Political Orientation

Using an adapted format (31), respondents were asked to report their political orientation on a 7-point scale ranging from “left” (coded as 1), “center” (coded as 4) to “right” (coded as 7) with an option of “I don't know” for those who did not position themselves in any of the political orientations. For ease of interpretation, responses were collapsed and recoded to three levels: “liberals” (strongly, moderately, or slightly left), “centrists” (center), and “conservatives” (slightly, moderately, or strongly right).

#### Demographic Variables

Information on sex, age, income and dwelling setting (urban, semi-urban, rural) was also collected.

### Data Analysis

By each level of policy support, two sets of 2 X 3 between-groups analysis of variance (ANOVA) were conducted to explore: (1) the impact of causes of inactivity (internal vs. external/both internal and external) and political orientation (liberals, centrists, conservatives) on support regarding policy action (low, moderately, and most intrusive policy actions); and, (2) the impact of responsibility for solutions (private matter vs. public health matter/both private and public health matter) and political orientation (liberals, centrists, and conservatives). Any significant interactions were followed-up with an analysis of simple effects (i.e., one-way ANOVA). *Post-hoc* testing using Bonferroni multiple comparison was conducted to further examine the differences in policy support by political orientation groups (liberals vs. centrists vs. conservatives).

## Results

With some overlap across these three items, respondents who answered “don't know” on their perceptions of the causes of physical inactivity (*n* = 101, 4.0%), or on their perception of the responsibility for solutions (*n* = 83, 3.5%), or who did not self-identify their political orientation (*n* = 376, 14.9%), were removed (*N* = 251). Thus, a total of 2044 participants (81.1% of recruited sample) were included in the final analyses. As compared to the Canadian population, the sample was well-distributed in terms of sex (male = 53.5%), age (*mean* = 50.2 ± 16.4 years, range 18-92), income (< $35,000 = 13.7%, ≤ $35,000 to <$75,000 = 29.0%, ≤ $75,000 to < $125,000 = 27.0%, > $125,000 = 14.6%, no response = 15.8%), and dwelling setting (urban = 54.2%, semi-urban = 26.8%, rural = 19.0%).

### Differences in Policy Support by Attributions for the Causes of Physical Inactivity and Political Orientation

Preliminary checks were conducted to ensure that no violations of the assumptions of normality, linearity, and homogeneity of variances existed (skewness between −0.12 and −0.91; kurtosis between −0.64 and 1.27). Though the ANOVA examining the effect of cause of inactivity and political orientation on policy support revealed no significant interaction on the least intrusive policy action support, *F*_(2, 2038)_ = 1.83, *p* = 0.16, the main effect of the cause *F*_(1, 2038)_ = 26.10, *p* <0.001, η^2^ = 0.013, was significant: those who held an internal causal attribution for physical inactivity showed less support for the least intrusive policy actions than those who held external causal attributions or both internal and external causal attributions. The main effects of political orientation, *F*_(2, 2038)_ = 44.86, *p* <0.001, η^2^ = 0.04, were also significant. *Post-hoc* test results revealed that conservatives showed less support than liberals, *p* < 0.001 or centrists, *p* < 0.001, and centrists showed less support than liberals, *p* < 0.05, for the least intrusive policy.

No significant interaction was detected between the cause of inactivity and political orientation on moderately intrusive policy support, *F*_(2, 2038)_ = 2.14, *p* = 0.12, but main effects of cause, *F*_(1, 2038)_ = 12.51, *p* <0.001, η^2^ = 0.006, were significant such that those who held an internal causal attribution showed less support for moderately intrusive policy actions than those who held both internal and external or external causal attributions. Significant main effects of political orientation, *F*_(2, 2038)_ = 25.36, *p* <0.001, η^2^ = 0.02, also were found. *Post-hoc* test results revealed that conservatives showed less support than liberals, *p* <0.001 or centrists, *p* <0.001, but no difference existed between liberals and centrists.

For the most intrusive policy actions, neither the interaction, *F*_(2, 2038)_ = 0.21, *p* = 0.81, nor the main effect of causal attribution were significant, *F*_(1, 2038)_ = 0.02, *p* = 0.88. No difference existed in support for intrusive policy approaches by causal attribution. However, the main effects of political orientation were significant, *F*_(2, 2038)_ = 33.19, *p* < 0.001, η^2^ = 0.03. For the most intrusive policies, conservatives showed less support than liberals, *p* < 0.001 or centrists, *p* < 0.001, and no difference was found between liberals and centrists. Individuals identifying as conservative showed the least support for the most intrusive policy actions regardless of their causal attribution for physical inactivity. The means and standard deviations for all policy supports by respondents' respondents' attributions for causes of physical inactivity and political orientation are described in Table 1.

**Table 1:** Means and standard deviations (SD) for public support on three types of policy by causal attribution and political orientation.

**Cause**	**Political orientation**	***N***	**Low intrusive**		**Moderately intrusive**		**Most intrusive**	
			**Mean**	***SD***	**Mean**	***SD***	**Mean**	***SD***
Only internal cause	Liberals	172	5.61	1.03	5.35	1.31	4.42	1.43
	Centrists	181	5.59	1.00	5.61	1.18	4.41	1.57
	Conservatives	252	5.08	1.10	4.96	1.44	3.80	1.61
	Total	605	5.38	1.08	5.26	1.36	4.16	1.58
Internal and external cause/Only external cause	Liberals	674	5.85	0.83	5.62	1.11	4.37	1.39
	Centrists	351	5.71	0.86	5.64	1.09	4.47	1.39
	Conservatives	414	5.42	0.93	5.29	1.35	3.82	1.53
	Total	1439	5.69	0.88	5.53	1.19	4.24	1.45
Total	Liberals	846	5.80	0.88	5.57	1.16	4.38	1.39
	Centrists	532	5.67	0.91	5.63	1.12	4.45	1.45
	Conservatives	666	5.29	1.01	5.16	1.39	3.81	1.56
	Total	2044	5.60	0.96	5.45	1.25	4.21	1.49

### Differences in Policy Support by Attributions for the Responsibility in Solving Physical Inactivity and Political Orientation

**Figure 1:**
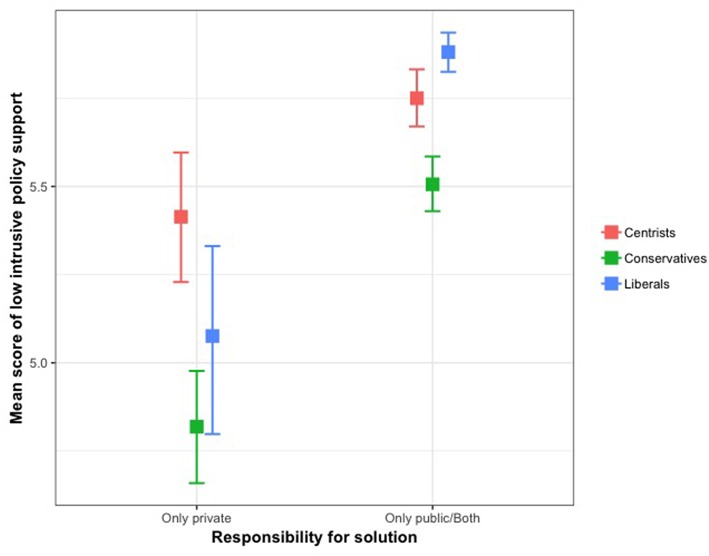
Association between responsibility for solutions and policital orientation on low intrusive policy support.

Since there was a significant interaction effect between responsibility and political orientation for the least intrusive policy support, *F*_(2, 2038)_ = 6.83, *p* < *0.0*1, η^2^ = 0.007, a series of simple test effects were conducted. There were significant differences in policy support among those who attributed physical inactivity as a private matter by individuals' political orientation, *F*_(2, 415)_ = 10.29, *p* < 0.001. Conservatives had less support than centrists, *p* < 0.001, but not compared to liberals (*p* > 0.05). Difference in policy support between liberals and centrists was not significant. Among those who attributed physical inactivity as both a private matter and public health matter or public health matter alone, differences in policy support by individuals' political orientation were found, *F*_(2, 1623)_ = 30.32, *p* < 0.001. Conservatives reported significantly less support than liberals, *p* < 0.001, and centrists, *p* < 0.001. Centrists also reported less support than liberals, *p* < 0.05. Figure 1 illustrates association between responsibility for solutions and political orientation on low intrusive policy support.

**Figure 2:**
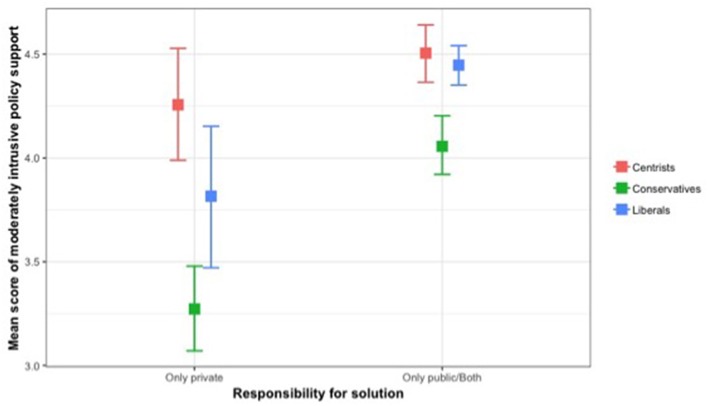
Association between responsibility for solutions and political orientation on moderately intrusive policy support.

For the moderately intrusive policy support, the interaction effect between responsibility for solutions and political orientation was significant, *F*_(2, 2038)_ = 3.53, *p* < 0.05, η^2^ = 0.003. A test of simple effects revealed significant differences in policy support by political orientation among those who attributed physical inactivity as a private matter, *F*_(2, 415)_ = 9.26, *p* < 0.001. Conservatives had lower support than centrists, *p* < 0.001. Conservatives also revealed less support than liberals, but the difference was not significant. Liberals reported less support than centrists, but the difference was not significant. Among those who attributed physical inactivity as both a private and public health matter or just a public health matter, significant differences existed in policy support by political orientation, *F*_(2, 1623)_ = 10.23, *p* < 0.001. Conservatives reported significantly less support than liberals, *p* < 0.01, and centrists, *p* < 0.001. The difference in support for the moderately intrusive policy between liberals and centrists was not significant. Figure 2 illustrates association between responsibility for solutions and political orientation on moderately intrusive policy support.

**Figure 3:**
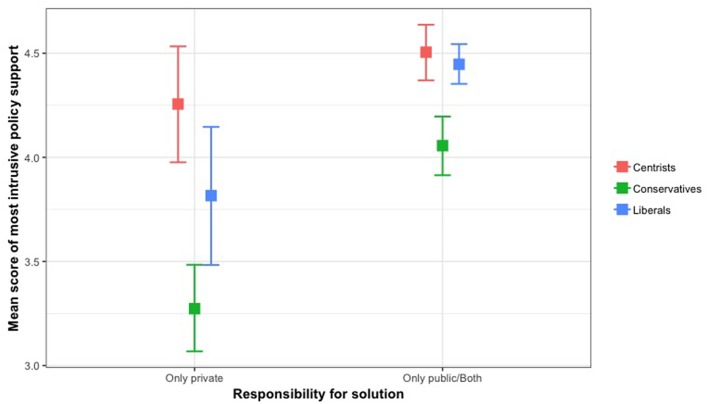
Association between responsibility for solutions and political orientation on most intrusive policy support.

For the most intrusive policy, the interaction effect between solution and political orientation was significant, *F*_(2, 2038)_ = 3.96, *p* < 0.05, η^2^ = 0.004. A test of simple effects revealed significant differences in policy support by political orientation among those who attributed physical inactivity as a private matter, *F*_(2, 415)_ = 16.10, *p* < 0.001. Conservatives reported less support than liberals, *p* < 0.05, and centrists, *p* < 0.001. The difference in the most intrusive policy support between liberals and centrists was not significant. Among those who attributed physical inactivity as both a private and public health matter or just a public health matter, differences in policy support by political orientation were also significant, *F*_(2, 1623)_ = 14.08, *p* < 0.001. Conservatives reported less support than liberals, *p* < 0.001, and centrists, *p* < 0.001. The difference in policy support between liberals and centrists was not significant. The means and standard deviations for all policy supports by respondents' attributions for the solutions of physical inactivity and political orientation are described in Table 2. Figure 3 illustrates association between responsibility for solutions and political orientation on most intrusive policy support.

**Table 2:** Means and standard deviations (SD) for public support on three types of policy by responsibility for solution and political orientation.

**Responsibility**	**Political orientation**	***N***	**Low intrusive**		**Moderately intrusive**		**Most intrusive**	
			**Mean**	***SD***	**Mean**	***SD***	**Mean**	***SD***
Only private	Liberals	87	5.08	1.25	4.93	1.51	3.82	1.61
	Centrists	125	5.41	1.07	5.38	1.25	4.26	1.56
	Conservatives	206	4.82	1.17	4.66	1.59	3.27	1.51
	Total	418	5.05	1.18	4.93	1.51	3.68	1.60
Private and public/Only public	Liberals	759	5.88	0.78	5.64	1.09	4.45	1.35
	Centrists	407	5.75	0.84	5.71	1.07	4.50	1.41
	Conservatives	460	5.51	0.85	5.39	1.23	4.06	1.52
	Total	1626	5.74	0.83	5.59	1.13	4.35	1.43
Total	Liberals	846	5.80	0.88	5.57	1.16	4.38	1.39
	Centrists	532	5.67	0.91	5.63	1.12	4.45	1.45
	Conservatives	666	5.29	1.01	5.16	1.39	3.81	1.56
	Total	2044	5.60	0.96	5.45	1.25	4.21	1.49

## Discussion

To the best of our knowledge, this is the first study to explore relationships between policy support, political orientation, and attributions for the causes of physical inactivity at a population level. Findings were mostly in-line with our hypotheses. First, those who attributed the cause of physical inactivity to internal causes reported less support for low and moderate intrusive policies compared to those who held external causal attributions or both internal and external causal attributions. However, only political orientation differentiated levels of support for the most intrusive policy actions. Unlike what was hypothesized, no interactions existed between causal attribution and political orientation on policy action support across all three level of intrusiveness. Previous studies examining the role of causal attribution in predicting public support of public health policy reported similar findings to the current study (21–23). It is notable the consistency in which causal attributions and political orientation distinguish acceptance of policies of varying levels of intrusiveness. Our findings demonstrate that such associations extend to physical inactivity although, in some cases, the effect sizes were small. In contrast, an interaction existed between responsibility for solutions and political orientation on support for policy actions to address physical inactivity. Conservatives who attributed responsibility for solving physical inactivity as a private matter had less support for all policy actions compared to centrists and liberals. This may be because responsibility for addressing public health issues directly implies potential actions of politicians and policy makers. Consequently, one's political orientation may be more salient in processing this question.

In the current study political orientation was more associated with policy support than causal attributions for physical inactivity. In other domains, individuals' internal causal attributions for obesity were negatively associated with policy support for obesity prevention whereas individuals' external or societal causal attribution were positively associated with support of societal actions including policy, legislation, and regulation (22, 30). With regard to respondents' political orientation, one experimental study in the United States (25) demonstrated that Republicans (conservatively oriented), in comparison with Democrats (liberally oriented), reported less policy support for public health policies after exposure to a news media article that described social determinants as the cause of type 2 diabetes; no differences in policy support by political orientation were found when the messages about the cause of type 2 diabetes were framed as a genetic or a behavioral factor. Similarly, conservative participants from the United States were opposed to the role of social determinants of health as well as government regulation that directly targeted an individual's choice (21). Rather, they emphasized individual responsibility for health and endorsed government responsibility in the promotion of health education. Yet, comparisons should be interpreted with caution as the health care system in the United States is dependent on the private sector in contrast to a publicly supported system in Canada.

The results of the current study confirm the importance of considering how individual health behaviors, like physical inactivity, are affected by broader social structures and social factors. Acknowledgment of these factors leads to the likely inevitable need to consider population level interventions (24). In which case, a more deliberative communication strategy may be the first step to enhance the receptiveness of the general public to increasingly intrusive policy actions across the political spectrum. Previous research in the context of obesity suggests the use of different images in messaging can influence support for policy between liberals and conservatives: the latter were more likely to support policy solutions for obesity when they viewed images of fast food (vs. images of overweight individuals), although they were less likely than liberal participants to support obesity prevention policies in general (37). Based on such findings with similar public health issues, future studies should examine how to best communicate and advocate to the public the need for an array of policy approaches addressing physical inactivity including those more intrusive in nature. For example, identifying an effective message that can combine collective responsibility approaches rather than overemphasizing either personal responsibility or societal responsibility may increase acceptance of government intrusion (38). This may involve framing messages that legislative policy action is not something that violates one's social beliefs and political views, but one that complements or even creates a synergy for individuals who are motivated to be more physically active by making active options the easiest options (38). Another option for connecting with more conservative perspectives is emphasizing potential benefits in reduction in health care costs through implementation of government actions to address physical inactivity. Giving greater prominence to the anticipated positive outcomes of potentially ‘unpopular' policy options may be critical (17).

Policy implementation is unlikely when the majority of the population is opposed to the initiative (39). This is particularly the case with policies that are more intrusive in nature, including new legislation and/or regulations. The effectiveness of policy actions generally increases as the level of intrusiveness increases, although public support is generally lower for interventions perceived as more intrusive (16, 17). This also varies by the target behavior, e.g., intrusive interventions restricting smoking in public places have been more supported than those addressing unhealthy diet and physical inactivity (16). Increasing awareness and understanding of the harms of second- and third-hand smoking has been used in communicating the need for legislative action in curbing smoking in public places (40). Whether an analogous external “harm” can be made for physical inactivity is unlikely although interesting models have been proposed regarding the socially contagious nature of obesity (41, 42). These models suggest that the obesity epidemic is different from traditional biological contagion that relies on spread by direct social contact. Rather, it spreads through the diffusion of unhealthy behaviors such as physical inactivity (41). For instance, friendship networks in schools influence the physical activity of individual children (43). Integrating such messaging, positively framed, within broader attempts to modify social norms as an intervention for promoting physical activity could be examined in future research and practice (44).

The current study is the first population-level examination of the association of perceptions of causal attribution of physical inactivity with different levels of policy support. Several limitations should be considered when interpreting the findings. First, the self-report nature of the cross-sectional design may be influenced by social desirability. Second, although items were adapted from previous studies, the measures have not been validated in a physical activity context. Furthermore, the internal consistency for the items categorized as the most intrusive policy was weak (Cronbach's α < 0.7). Third, other factors may affect respondents' policy support. For example, respondents' health status (27) and health literacy (9, 45) may be influential. Last, as acknowledged in a previous study (28), comparing and contrasting public support of policy interventions for a range of different chronic disease risk factors such as alcohol use or healthy diets would provide more meaningful implications for policy makers and public health promoters in terms of prioritizing and concurrently implementing policy actions across a range of public health issues (28). This represents an important future research direction.

## Conclusion

Physical inactivity is the most common lifestyle risk factor contributing to premature morbidity and mortality in Canada (46). Our results suggest that public acceptance of policy actions addressing physical inactivity will vary by the attributions the public have regarding its causes and solutions, and by political orientation. Advocacy and messaging for progressive policy implementation in the physical activity arena will need to navigate this terrain and communicate in ways that encourage reflective and informed deliberation (47) that is inclusive of the broad spectrum of the Canadian population.

## Data Availability

The datasets generated for this study are available on request to the corresponding author.

## Ethics Statement

ParticipACTION, a Canadian non-profit organization promoting physical activity (www.participaction.com), commissioned the original survey as part of its ongoing public relations and advocacy work. Specifically, a total sample was recruited from an online panel collected via a Canadian survey firm (Angus Reid Forum). By enrolling as a panelist in the Angus Reid Forum, recruited individuals consented to their participation in invited surveys or panel discussions. Given the secondary analyses undertaken in this study were of minimal risk and utilized anonymous data, ethical approval was not needed in accordance with article 2.4 and 5.5 of the Canadian Tri-Council policy statement (TCPS2) regarding ethical conduct of human research^1^.

## Author Contributions

LY performed data analysis and prepared the original manuscript. TB, AL-C, MT, JS, RR, and NO provided input for the development of the survey, commented on the research method and contributed to manuscript edits. LV managed the data collection process and contributed to manuscript edits. GF oversaw a whole research process as the supervisory author. All authors read and approved final manuscript.

### Conflict of Interest Statement

GF, TB, AL-C, MT, JS, RR, and NO are members of ParticipACTION's volunteer-based Research Advisory Group, and LV works as ParticipACTION's Knowledge Translation Manager. No financial or promotional advantages were gained from conducting this study. The remaining author declares that the research was conducted in the absence of any commercial or financial relationships that could be construed as a potential conflict of interest.

## Footnotes

^1^Canadian Institutes of Health Research, Natural Sciences and Engineering Research Council of Canada, and Social Sciences and Humanities Research Council of Canada, Tri-Council Policy Statement: Ethical Conduct for Research Involving Humans. http://www.pre.ethics.gc.ca/pdf/eng/tcps2-2014/TCPS_2_FINAL_Web.pdf. Published December 2014. Accessed December 05, 2018.

## References

[1] Warburton DE Charlesworth S Ivey A Nettlefold L Bredin SS. A systematic review of the evidence for Canada's Physical Activity guidelines for adults. Int J Behav Nutr Phys Act. (2010) 7:39. 10.1186/1479-5868-7-39 20459783 PMC3583166

[2] Lubans D Richards J Hillman C Faulkner G Beauchamp M Nilsson M. Physical activity for cognitive and mental health in youth: a systematic review of mechanisms. Pediatrics. (2016) 138:1–15. 10.1542/peds.2016-1642 27542849

[3] World Health Organization Global Action Plan on Physical Activity 2018–2030: More Active People for a Healthier World. Geneva (2018). Available online at: https://www.who.int/ncds/prevention/physical-activity/global-action-plan-2018-2030/en/ (accessed December 5, 2018).

[4] Ding D Lawson KD Kolbe-Alexandar TL Finkelstein EA Katzmarzyk PT Mechelen W. The economic burden of physical inactivity: a global analysis of major non-communicable diseases. Lancet. (2016) 388:1311–24. 10.1016/S0140-6736(16)30383-X 27475266

[5] Bounajm F Dinh T Theriault L Moving Ahead: The Economic Impact of Reducing Physical Inactivity and Sedentary Behaviour. Ottawa: The Conference Board of Canada (2014). Available online at: https://www.conferenceboard.ca/temp/40939ef1-9874-4923-8582-4e911962b49b/6436_MovingAheadRPI-BR(PUB3386).pdf (accessed December 5, 2018).

[6] Bull FC Bauman AE. Physical inactivity: the Cinderella risk factor for noncommunicable disease prevention. J Health Commun. (2011) 16:13–26. 10.1080/10810730.2011.601226 21916710

[7] Chung-Hall J Craig L Driezen P Ouimet J Sansone G Fong GT Canadian Smokers' Support for Tobacco Endgame Strategies: Findings from the ITC Canada Survey. Waterloo, ON: University of Waterloo (2016). Available online at: www.itcproject.org/files/ITC_Endgame_Report_26_Sept2016_FINAL.pdf (accessed December 5, 2018).

[8] Health Canada Seizing the Opportunity: The Future of Tobacco Control in Canada. Ottawa, ON (2017). Available online at: https://www.canada.ca/en/health-canada/programs/future-tobacco-control/future-tobacco-control.html (accessed December 5, 2018).

[9] Bhawra J Reid JL White CM Vanderlee L Raine K Hammond D. Are young Canadians supportive of proposed nutrition policies and regulations? An overview of policy support and the impact of socio-demographic factors on public opinion Can J Publ Health. (2018) 109:498–505. 10.17269/s41997-018-0066-1 29981092 PMC6964476

[10] Senate of Canada Obesity in Canada: a Whole-of-Society Approach for a Healthier Canada. Ottawa, ON: The Standing Senate Committee on Social Affairs (2016). Available online at: https://sencanada.ca/content/sen/committee/421/SOCI/Reports/2016-02-25_Revised_report_Obesity_in_Canada_e.pdf (accessed December 5, 2018).

[11] Spence JC Faulkner G Costas-Bradstreet C Duggan M Tremblay MS. Active Canada 20/20: a physical activity plan for Canada. Can J Publ Health. (2015) 106:e470–e473. 10.17269/CJPH.106.5041 26986905 PMC6972327

[12] Craig CL. Evolution and devolution of national physical activity policy in Canada. J Phys Act Health. (2011) 8:1044–56. 10.1123/jpah.8.8.1044 22039123

[13] Public Helath Agency of Canada A Common Vision for Increasing Physical Activity and Reducing Sedentary Living in Canada: Let's Get Moving. Ottawa, ON. (2018). Available online at: https://www.canada.ca/en/public-health/services/publications/healthy-living/lets-get-moving.html (accessed December 5, 2018).

[14] Li J Lovatt M Eadie D Dobbie F Meier P Holmes J. Public attitudes towards alcohol control policies in Scotland and England: results from a mixed-methods study. Soc Sci Med. (2017) 177:177–89. 10.1016/j.socscimed.2017.01.037 28171817 PMC5341733

[15] Nutfield Council on Bioethics Policy Process and Practice. London: Public Health (2007) p. 29-48. Available online at: http://nuffieldbioethics.org/wp-content/uploads/2014/07/Public-health-Chapter-3-Policy-process-and-practice.pdf (accessed December 5, 2018).

[16] Diepeveen S Ling T Suhrcke M Roland M Marteau TM. Public acceptability of government intervention to change health-related behaviours: a systematic review and narrative synthesis. BMC Publ Health. (2013) 13:756. 10.1186/1471-2458-13-756 23947336 PMC3765153

[17] Pechey R Burge P Mentzakis E Suhrcke M Marteau TM. Public acceptability of population-level interventions to reduce alcohol consumption: A discrete choice experiment. Soc Sci Med. (2014) 113:104–9. 10.1016/j.socscimed.2014.05.010 24858928 PMC4065329

[18] Magnusson RS. Case studies in nanny state name-calling: what can we learn? Publ Health. (2015) 129:1074–82. 10.1016/j.puhe.2015.04.023 26074167

[19] Weiner B. An attributional theory of achievement motivation and emotion. Psychol Rev. (1985) 92:548–73. 3903815

[20] Weiner B Social Motivation, Justice, and the Moral Emotions: Anattributional Approach. Mahwah, NJ: Lawrence Erlbaum Associates (2006). Available online at: https://samarnhpang.files.wordpress.com/2011/06/social-justice-and-learning.pdf (accessed December 5, 2018).

[21] Lundell H Niederdeppe J Clarke C. Public views about health causation, attributions of responsibility, and inequality. J Health Commun. (2013) 18:1116–1130. 10.1080/10810730.2013.768724 23679219

[22] McGlynn J McGlone MS Desire or disease? framing obesity to influence attributions of responsibility and policy support. Health Commun. (2018) 34:689–701. 10.1080/10410236.2018.1431025 29388801

[23] Ortiz SE Zimmerman FJ Adler GJ Jr. Increasing public support for food-industry related, obesity prevention policies: The role of a taste-engineering frame and contextualized values. Soc Science Med. (2016) 156:142–153. 10.1016/j.socscimed.2016.02.042 27038322

[24] Robert SA Booske BC. US opinions on health determinants and social policy as health policy. Am J Publ Health. (2011) 101:1655–63. 10.2105/AJPH.2011.300217 21778491 PMC3154244

[25] Gollust SE Lantz PM Ubel PA. The polarizing effect of news media messages about the social determinants of health. Am J Publ Health. (2009) 99:2160–7. 10.2105/AJPH.2009.161414 19833981 PMC2775784

[26] Bobbio A Canova L Manganelli AM Conservative ideology, economic conservatism, and causal attributions for poverty and wealth. Curr Psychol. (2010) 29:222–34. 10.1007/s12144-010-9086-6

[27] LeCount RJ Abrahamson K Self-rated health and attitudes about US health care policy. Sociol Spectr. (2017) 37:237–49. 10.1080/02732173.2017.1334607

[28] Ashley MJ Cohen JE Northrup DA Ferrence RG. How canadian legislators view health promotion: does party affiliation matter? Can J Publ Health. (2001) 92:16–18. Available online at: https://www.jstor.org/stable/41993257 11257983 10.1007/BF03404836 PMC6980111

[29] Yun L Vanderloo L Berry TR Latimer-Cheung AE O'Reilly N Rhodes RE. Assessing the social climate of physical (in)activity in Canada. BMC Publ Health. (2018) 18:1301. 10.1186/s12889-018-6166-2 30482164 PMC6258462

[30] Oliver JE Lee T. Public opinion and the politics of obesity in America. J Health Polit Policy Law. (2005) 30:923–54. 10.1215/03616878-30-5-923 16477792

[31] Gollust SE Niederdeppe J Barry CL. Framing the consequences of childhood obesity to increase public support for obesity prevention policy. Am J Publ Health. (2013) 103:e96–e102. 10.2105/AJPH.2013.301271 24028237 PMC3828688

[32] Canadian Institutes of Health Research Natural Sciences and Engineering Research Council of Canada and Social Sciences and Humanities Research Council of Canada Tri-Council Policy Statement: Ethical Conduct of Research Involving Humans. Ottawa, ON (2010). Available online at: http://www.pre.ethics.gc.ca/pdf/eng/tcps2-2014/TCPS_2_FINAL_Web.pdf (assessed December 5, 2018).

[33] Raine KD Nykiforuk CI Vu-Nguyen K Nieuwendyk LM VanSpronsen E Reed S. Understanding key influencers' attitudes and beliefs about healthy public policy change for obesity prevention. Obesity. (2014) 22:2426–33. 10.1002/oby.20860 25131938

[34] Cohen JE Nicole A Ashley MJ Ferrence R Northrup DA Studlar DT. Predictors of Canadian legislators' support for tobacco control policies. Soc Sci Med. (2002) 55:1069–1076. 10.1016/S0277-9536(01)00244-1 12220090

[35] The International Tobacco Control ITC Canada National Report. Findings from the Wave 1 to 8 Surveys (2002-2011). Waterloo, ON. (2013). Available online at: https://www.itcproject.org/files/ITC_Canada_Report-English-Nov6-2014v16-print.pdf (accessed December 5, 2018).

[36] Sikorski C Luppa M Brähler E König H-H Riedel-Heller SG. Obese children, adults and senior citizens in the eyes of the general public: results of a representative study on stigma and causation of obesity. PLoS ONE. (2012) 7:e46924. 10.1371/journal.pone.0046924 23071664 PMC3470564

[37] Young R Hinnant A Leshner G. Individual and social determinants of obesity in strategic health messages: Interaction with political ideology. Health Commun. (2016) 31:903–10. 10.1080/10410236.2015.1018699 26698295

[38] Brownell KD Kersh R Ludwig DS Post RC Puhl RM Schwartz MB. Personal responsibility and obesity: a constructive approach to a controversial issue. Health Aff. (2010) 29:379–87. 10.1377/hlthaff.2009.0739 20194976

[39] Blendon RJ Hunt K Benson JM Fleischfresser C Buhr T. Understanding the American public's health priorities: a 2006 perspective. Health Aff. (2006) 25:w508-w515. 10.1377/hlthaff.25.w508 17046841

[40] Fahy D Trench B Clancy L. Communicating contentious health policy: lessons from Ireland's workplace smoking ban. Health Promot Pract. (2012) 13:331–8. 10.1177/1524839909341554 19815655

[41] Huang H Yan Z Chen Y Liu F. A social contagious model of the obesity epidemic. Sci Rep. (2016) 6:1–9. 10.1038/srep37961 27892501 PMC5124998

[42] Christakis NA Fowler JH. The spread of obesity in a large social network over 32 years. N Engl J Med. (2007) 357:370–9. 10.1056/NEJMsa066082 17652652

[43] Stearns JA Godley J Veugelers PJ Ekwaru JP Bastian K Wu B. Associations of friendship and children's physical activity during and outside of school: a social network study. SSM Popul Health. (2019) 7:100308. 10.1016/j.ssmph.2018.10.008 30581958 PMC6288406

[44] Ball K Jeffery RW Abbott G McNaughton SA Crawford D. Is healthy behavior contagious: associations of social norms with physical activity and healthy eating. Int J Behav Nutr Phys Act. (2010) 7:86. 10.1186/1479-5868-7-86 21138550 PMC3018448

[45] Spence JC Brawley LR Craig CL Plotnikoff RC Tremblay MS Bauman A. ParticipACTION: Awareness of the participACTION campaign among Canadian adults-Examining the knowledge gap hypothesis and a hierarchy-of-effects model. Int J Behav Nutr Phys Act. (2009) 6:1. 10.1186/1479-5868-6-85 19995456 PMC2795738

[46] Manuel DG Perez R Sanmartin C Taljaard M Hennessy D Wilson K. Measuring burden of unhealthy behaviours using a multivariable predictive approach: life expectancy lost in Canada attributable to smoking, alcohol, physical inactivity, and diet. PLoS Med. (2016) 13:e1002082. 10.1371/journal.pmed.1002082 27529741 PMC4986987

[47] Grunseit AC Rowbotham S Crane M Indig D Bauman AE Wilson A Nanny or canny? Community perceptions of government intervention for preventive health. Crit Publ Health. (2018) 29:1–16. 10.1080/09581596.2018.1468020

