# Supplementary material for: Political Orientation and Public Attributions for the Causes and Solutions of Physical Inactivity in Canada: Implications for Policy Support
Source: Front Public Health. 2019 Jun 18;7:153. doi: 10.3389/fpubh.2019.00153 (PMC6611409; doi:10.3389/fpubh.2019.00153)
Supplement: Supplementary file 1 [file Table_1.DOCX]

**Electronic Supplementary Materials**

Table 1. Principal Component Analysis Result - Total Variance Explained

|  | Initial Eigenvalues | | | Extraction Sums of Squared Loadings | | | Rotation Sums of Squared Loadings |
| --- | --- | --- | --- | --- | --- | --- | --- |
| Component | Total | % of Variance | Cumulative % | Total | % of Variance | Cumulative % | Total |
| 1 | 6.75 | 39.69 | 39.69 | 6.75 | 39.69 | 39.69 | 6.02 |
| 2 | 1.29 | 7.58 | 47.28 | 1.29 | 7.58 | 47.28 | 3.67 |
| 3 | 1.25 | 7.37 | 54.64 | 1.25 | 7.37 | 54.64 | 3.24 |

1. Low intrusive policy actions

2. Moderately intrusive policy actions

3. Most intrusive policy actions

Table 2. Component Matrix

|  | Component | | |
| --- | --- | --- | --- |
|  | 1  Low Intrusive | 2  Moderately Intrusive | 3  Most Intrusive |
| Item1: Provide programs to educate, inspire, support, or motivate the general public about the importance of regular physical activity | 0.75 |  |  |
| Item 2: Fund media campaigns to educate the public about increasing physical activity and reducing screen time | 0.71 |  |  |
| Item 3: Create and share guidelines for adults that provide guidance on physical activity, sedentary behavior and sleep | 0.70 |  |  |
| Item 4: Increase training of educators and school support staff to deliver quality physical activity programming | 0.64 | -0.36 |  |
| Item 5: Enhance the quantity and quality of green spaces in all neighbourhoods | 0.70 |  |  |
| Item 6: Implement transportation policies designed to promote physical activity through safe routes, cycle facilities, adequate lighting, etc. | 0.73 |  |  |
| Item 7: Change the design of our neighbourhoods and communities to encourage informal physical activity in daily life | 0.71 |  |  |
| Item 8: Provide support to guarantee safe and supported play areas in urban environments (e.g., security/chaperone at an urban basketball court) | 0.68 |  |  |
| Item 9: Improve universal accessibility (e.g., wheelchair access) of recreation facilities to enable participation among all ability groups | 0.63 | -0.33 |  |
| Item 10: Provide mandatory daily physical education or physical activity requirements in all schools | 0.50 |  |  |
| Item 11: Provide incentives for workplaces to develop physical activity policies and access to physical activity facilities for workers | 0.68 |  |  |
| Item 12: Subsidize programs that encourage people to be physically active | 0.69 |  |  |
| Item 13: Remove sales taxes on all physical activity equipment | 0.46 | 0.38 | -0.48 |
| Item 14: Provide tax credits or monetary incentives for people who are involved in physical activity | 0.55 | 0.55 | -0.37 |
| Item 15: Ban all traffic in high-use pedestrian areas during peak hours to support active (walking, cycling) or public transportation | 0.55 |  | 0.54 |
| Item 16: Restrict the use of elevators for trips three floors or less (e.g. exceptions include use by individuals with disabilities, persons with baby strollers) | 0.46 | 0.36 | 0.51 |
| Item 17: Redirect government funding for high performance sport (e.g., Olympians) to recreational sport | 0.44 | 0.33 |  |

Table 3. Pattern Matrix

|  | Component | | |
| --- | --- | --- | --- |
|  | 1  Low Intrusive | 2  Moderately Intrusive | 3  Most Intrusive |
| Item1: Provide programs to educate, inspire, support, or motivate the general public about the importance of regular physical activity | 0.69 |  |  |
| Item 2: Fund media campaigns to educate the public about increasing physical activity and reducing screen time | 0.54 |  |  |
| Item 3: Create and share guidelines for adults that provide guidance on physical activity, sedentary behavior and sleep | 0.69 |  |  |
| Item 4: Increase training of educators and school support staff to deliver quality physical activity programming | 0.80 |  |  |
| Item 5: Enhance the quantity and quality of green spaces in all neighbourhoods | 0.70 |  |  |
| Item 6: Implement transportation policies designed to promote physical activity through safe routes, cycle facilities, adequate lighting, etc. | 0.58 |  |  |
| Item 7: Change the design of our neighbourhoods and communities to encourage informal physical activity in daily life | 0.52 |  |  |
| Item 8: Provide support to guarantee safe and supported play areas in urban environments (e.g., security/chaperone at an urban basketball court). | 0.63 |  |  |
| Item 9: Improve universal accessibility (e.g., wheelchair access) of recreation facilities to enable participation among all ability groups | 0.76 |  |  |
| Item 10: Provide mandatory daily physical education or physical activity requirements in all schools | 0.59 |  |  |
| Item 11: Provide incentives for workplaces to develop physical activity policies and access to physical activity facilities for workers |  | 0.46 |  |
| Item 12: Subsidize programs that encourage people to be physically active |  | 0.59 |  |
| Item 13: Remove sales taxes on all physical activity equipment |  | 0.79 |  |
| Item 14: Provide tax credits or monetary incentives for people who are involved in physical activity |  | 0.87 |  |
| Item 15: Ban all traffic in high-use pedestrian areas during peak hours to support active (walking, cycling) or public transportation |  |  | 0.76 |
| Item 16: Restrict the use of elevators for trips three floors or less (e.g. exceptions include use by individuals with disabilities, persons with baby strollers) |  |  | 0.78 |
| Item 17: Redirect government funding for high performance sport (e.g., Olympians) to recreational sport |  |  | 0.51 |
